# Forecasting the seasonal phenology of *Agrotis ipsilon* in Oregon grass seed and vegetable agroecosystems

**DOI:** 10.3389/finsc.2024.1505524

**Published:** 2025-01-17

**Authors:** Emma Slone, Jessica Green, Navneet Kaur, Darrin L. Walenta, Nicole P. Anderson, Casey Cruse, Seth J. Dorman

**Affiliations:** ^1^ USDA-ARS, Forage Seed and Cereal Research Unit, Corvallis, OR, United States; ^2^ Department of Environmental and Molecular Toxicology, Oregon State University, Corvallis, OR, United States; ^3^ Department of Crop and Soil Science, Oregon State University, Corvallis, OR, United States; ^4^ Division of Food Production and Society, Norwegian Institute of Bioeconomy Research (NIBIO), Ås, Norway

**Keywords:** black cutworm, *lolium perenne*, pest forecasts, phenological modeling, temperaturedependent development

## Abstract

*Agrotis ipsilon* (Lepidoptera: Noctuidae) is a significant pest in Oregon grass seed and vegetable production systems. Effective management of this species relies on timely foliar insecticide applications targeting immature *A. ipsilon* larvae before crop damage is observed. Regionally specific phenological models serve as a critical component of effective areawide pest management plans to inform the timing of pest monitoring and management action. Seasonal modeling of *A. ipsilon* phenology is complicated by their migratory behavior and limited knowledge of temperature-dependent development on affected crop hosts. Growth chamber experiments at five constant temperatures (12 to 32°C) were conducted to determine the temperature-dependent development of *A. ispsilon* life stages on an artificial and perennial ryegrass diet. The completion of one *A. ipsilon* generation (egg-to-adult) required 658.71 ± 31.49, 601.98 ± 16.01, 648.47 ± 21.35 degree days with a base temperature threshold of 9.8°C for artificial diet, perennial ryegrass diet, and across both diet types, respectively. The timing of migrant adults was predicted with surface air temperature using non-linear regression with *A. ipsilon* abundance data collected from pheromone-baited traps in 77 total commercial grass seed (n = 57) and vegetable (n = 20) production fields across 19 sampling years (1996 to 2023). Developmental parameters and predictions of adult arrival were used to develop general and grass seed specific phenology model projections for *A. ipsilon* populations in Oregon. Regionally validated phenology models can be incorporated into decision support tools to forecast the spatiotemporal occurrence of crop-damaging life stages of priority insect pests.

## 1 Introduction

Migratory moth pests within the Noctuidae family pose a substantial threat to agriculture production globally due, in part, to their strong flight capability, polyphagous feeding behavior, broad host range, and multivoltine life history (1, 2, 38). Synthetic pesticides are widely utilized for pest control worldwide (>3 Tg of pesticides are applied annually) and are a primary strategy for managing noctuid pests (3). An array of factors contribute to insecticide resistance evolution, including pesticide-based programs that rely on excessive applications of broad-spectrum insecticides and limited rotations with other modes of action (MoAs). These factors often lead to more frequent insecticide applications and the need to register new MoAs when high levels of insecticide resistance are selected for in target pest populations, a process colloquially known as the ‘pesticide treadmill’ (4). Pest management strategies employed within an areawide framework may offer compounded benefits regarding pest suppression and insecticide resistance management by reducing regional population densities of highly mobile pests, lowering reliance on chemical inputs for crop protection (5–8). A key tenet of effective areawide pest suppression and insecticide resistance management involves pest surveillance to determine when populations are present at susceptible life stage(s) and above economically damaging levels, enabling appropriately timed management action (9). Regionally validated phenology models can inform decision support platforms that predict the timing of susceptible pest life stages across broad spatial scales (10). Furthermore, spatialized phenological predictions using real-time environmental data can increase the resolution, efficiency, and usability of pest forecasting tools for crop management implementers (11, 12). Range expansion and phenological shifts of migratory species fueled by climate change have further intensified the need for precise spatiotemporal risk assessments of priority pests (13–15).

*Agrotis ipsilon*, commonly known as the black cutworm or greasy cutworm, is a significant noctuid pest in temperate agricultural systems worldwide (38). Similar to other prolific migratory noctuid pests, *A. ipsilon* larvae have a wide host range across nine plant families, including agronomic crops maize, tobacco, cotton, wheat, vegetables, turf grasses used for grazing, and grasses grown for seed (16). *Agrotis ipsilon* inflicts the most severe crop damage by severing stems of young seedlings at or below soil level and feeding on roots as well as significant crop defoliation, leading to economic reductions in yield potential when large-scale infestations occur (38). *Agrotis ipsilon* adults migrate from southern latitudes, reaching central and western Oregon in late spring. Migrant adults produce at least one complete generation in Oregon; subsequent adults then emigrate southward in the autumn to evade unfavorable freezing temperatures. Chemical control is the primary strategy for managing *A. ipsilon* in vegetable and grass seed cropping systems in Oregon, which is complicated by the subterranean and nocturnal behavior of the crop-damaging larval life stage that burrows into the soil during the day (~2 to 5 cm), only surfacing at night and on overcast days. Foliar insecticide applications are most effective in targeting immature larvae (first to third instar) when larvae are most susceptible to insecticides and before severe crop damage is observed. Precise knowledge of the space-time phenological patterns of vulnerable life stages of *A. ipsilon* in Oregon vegetable crops and grasses grown for seed is needed to promote areawide integrated pest management (IPM) practices through improved timing and allocation of pest monitoring and management resources. Moreover, climate change will undoubtedly contribute to changes in the seasonal phenology of insect pests, further accentuating the need for regionally accurate phenology models (17).

Insects exhibit a positive correlation between their rate of development and increasing temperature. Insect phenology and temperature-dependent development vary regionally based on local weather patterns and crop hosts (18). The relationship between temperature and insect phenology is often used to develop degree-day models that predict insect life-stages utilizing daily heat summations and lower and upper developmental thresholds (19). Previous studies investigating *A. ipsilon* development have focused exclusively on *A. ipsilon* reared on artificial diets or maize (16, 20). Understanding *A. ipsilon’s* developmental timeline on grass seed crops, a primary host with the greatest land area across the Willamette Valley compared to other *A. ipsilion* crop hosts, may be critical for developing regionally specific *A. ipsilon* phenology models utilizing heat summation calculations. As such, the objectives of this study were two-fold: 1) determine the temperature-dependent development of *A. ipsilon* reared on two distinct diet types, including perennial ryegrass (*Lolium perenne*) and an artificial diet at five constant temperatures from 12 to 32°C, and 2) predict the timing of median adult *A. ipsilon* flight in Oregon commercial grass seed and vegetable crops using pheromone baited trap networks deployed from 1996 to 2023 to inform the biofix date needed to initiate degree day accumulation for life stage thresholds. Research findings will inform model parameters to predict the phenology of *A. ipsilon* in Oregon and enable areawide decision support tool development.

## 2 Methods

### 2.1 Temperature-dependent development

#### 2.1.1 Larval and pupal development

To evaluate the temperature-dependent development of *A. ipsilon*, an established laboratory colony was maintained at 22 ± 1°C, 60 ± 5% RH, and 16L:8D photoperiod. Neonate larvae, within 24 h from egg hatch, were placed in individual incubators (Percival^®^ model DR-36VL) set to one of five constant temperatures, including 12, 17, 22, 27, and 32°C (60 ± 5% RH and 16L:8D photoperiod) based on previous temperature-dependent studies on *A. ipsilon* and related species (20, 21). Two diet treatments were evaluated at each constant temperature (n = 100 total larvae per diet type; n = 20 larvae per temperature and diet treatment combination), including an artificial diet (soy wheat-germ diet) and a perennial ryegrass diet (cultivar Fastball 3GL) consisting of fresh grass tissue clippings. For both diet types, enough artificial diet (approximately 0.7 g) or grass clippings (approximately 1.2 g) were placed in 1 oz plastic cups to cover the bottom of the cup. Single neonate larvae were confined in individual cups (one specimen per cup) with aerated lids wrapped with thrips screen (150 ×150 μm) to prevent escape. Temperature and RH were recorded in 30-minute intervals with two iButton^®^ data loggers (Thermochron Temperature Loggers; Sydney, Australia) placed in additional lidded plastic cups with both artificial diet and grass seed diet types to replicate the test environment. Diet was replaced at least three times per week, and data was recorded daily. Data collection for the larval life stage included the date, current larval instar (evaluated based on exuviae and head capsule width), and mortality if present. Larvae were allowed to pupate in 1 oz plastic cups; the date of pupation was recorded, and pupae were sexed before adult emergence.

#### 2.1.2 Adult and egg development

Following adult emergence, individual moths were placed in nylon mesh enclosures (23 × 23 × 28 cm) and fed a 10% sucrose solution dispensed on a cotton ball. The temperature, RH, and photoperiod regimen remained the same as previously described for larval and pupal development. When female and male adults had emerged, single female-male pairs were placed in one enclosure. Strips of nylon mesh and paper were affixed to the top of the enclosure to serve as an oviposition substrate. The date of oviposition, adult mortality, total number of eggs, and subsequent egg hatch (number of viable eggs hatched per matriline) were recorded daily.

### 2.2 Adult flight phenology and biofix prediction

**Figure 1:**
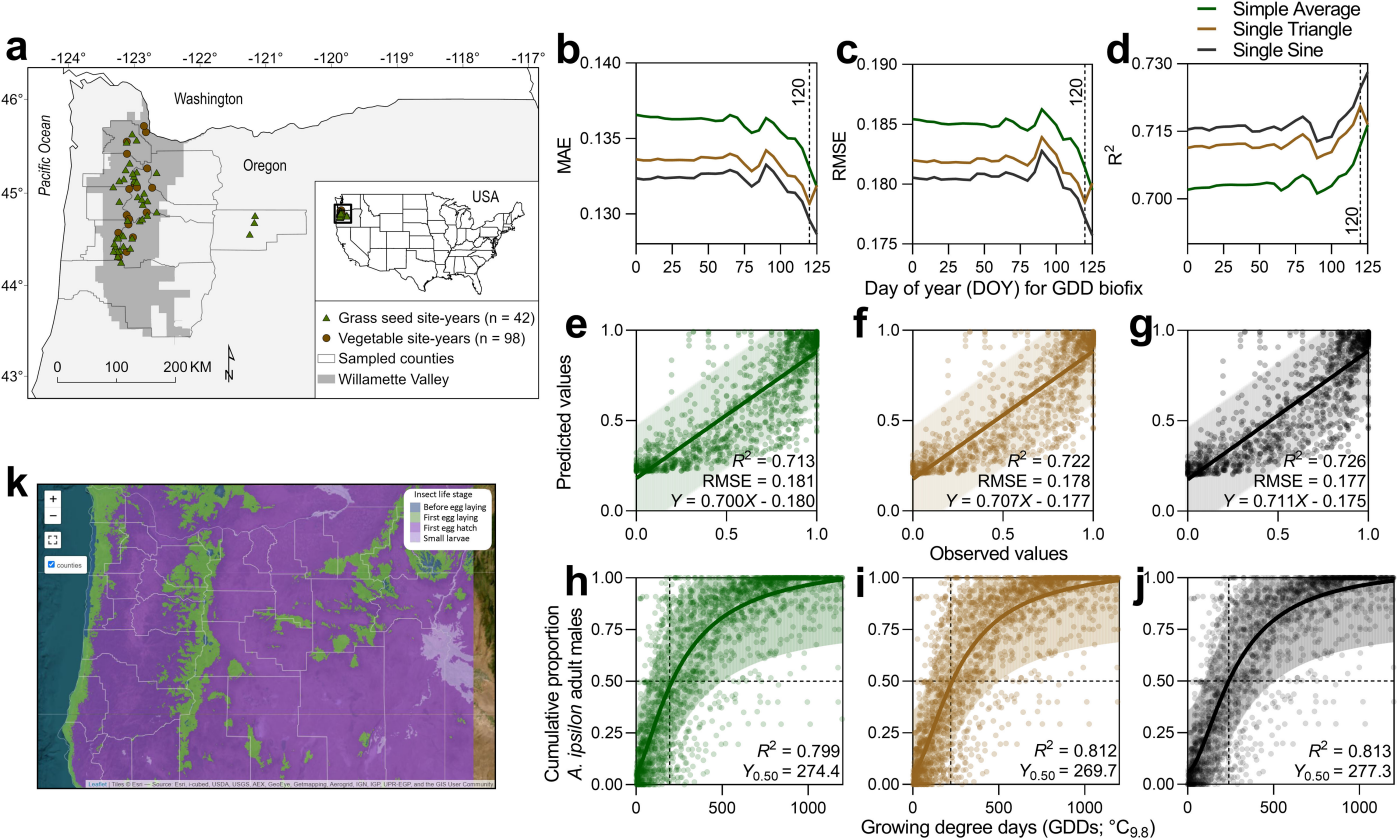
Site-year locations of *A. ipsilon* pheromone-baited traps in vegetable (n = 98) and grass seed crops (n = 42) from 1996 to 2023 **(A)**. Model fit metric comparisons, including MAE **(B)**, RMSE **(C)**, and R^2^ **(D)**, to determine the optimal start date for degree day accumulation for predicting *A*. *ipsilon* median flight. Linear regression model comparisons between observed and predicted values for *A. ipsilon* flight using three degree day calculation methods, including simple average **(E)**, single triangulation **(F)**, and single-sine **(G)**. Nonlinear regression model output and thermal requirements of predicted *A. ipsilon* median flight using simple average **(H)**, single triangulation **(I)**, and single-sine **(J)** degree day calculation methods (degree day accumulation start date = DOY 120, LDT = 9.8°C). *Agrotis ipsilon* phenology forecasts incorporated into a real-time decision support platform (**K**; https://oregonpests-usdaars.hub.arcgis.com).

To predict *A. ipsilon* flight phenology and determine the biofix date for degree day accumulation, an attract-based trap network was deployed in commercial fields, including vegetable (sweet corn, broccoli, snap bean) and grass seed crops (perennial ryegrass, tall fescue, Kentucky bluegrass) across the Willamette Valley and central Oregon. A total of 17 unique locations were selected for monitoring in vegetable crops across 15 years from 1996 to 2011 (n = 98 site-years) and 29 locations in grass seed crops across two years (n = 42 site-years) (**Figure 1A**). *Agrotis ipsilon* adults were monitored weekly for a minimum of ten consecutive weeks from May through October. Adult abundance was quantified using one green funnel trap (Unitrap™) or Hartstack trap baited with female sex pheromone lure (Z9-14 Ac and Z7-12Ac; Alpha Scents Inc., Canby, Oregon) per field, positioned >15 m from field borders. Pheromone lures and insecticidal pest strips (10% dichlorvos active ingredient) placed in traps to prevent escape were replaced every three weeks. Collected specimens were transferred to the laboratory to confirm idenficiation using Leica S9i stereomicroscopes (Leica Microsystems, Wetzlar, Germany). Traps deployed in commercial vegetable and grass seed crops were monitored for an average of 19.9 ± 0.6 and 17.4 ± 0.6 consecutive weeks, respectively (hereafter, ‘mean ± SE’).

### 2.3 Data analysis

#### 2.3.1 Temperature-dependent development

All data analyses were performed in R (v4.3.1 R Core Team). Differences in development time (number of days) for each life stage were evaluated independently across temperature and diet treatments using linear mixed models (LMMs) with temperature treatment and diet as fixed effects and random effects intercepts for specimen replicate using the *lme4* package (22). The *emmeans* package (23) was used to estimate estimated marginal means from LMMs and compared using Tukey’s HSD test (α = 0.05 significance level) to determine treatment differences.

The temperature-dependent development rate for each life stage by diet type was determined using simple linear regression; development rates were also evaluated for combined data across diet type (hereafter, ‘combined’ dataset). The reciprocals (*y*) of the number of days of development for each life stage were determined for each specimen replicate and plotted against the constant temperature treatment (*T*) (i.e., *y* = *a* + *bT*). As described by Campbell et al. (24), the lower temperature threshold *t* and thermal constant *k* (number of degree days) were calculated as $t\;=\;\frac{-a}{b}$ and $k\;=\;\frac{1}{b}$ where *a* and *b* are the intercept and slope of the regression line, respectively. The standard errors (hereafter, ‘SE’) for *t* were calculated as


$$\frac{\bar{y}}{b}\;\sqrt{\frac{s^{2}}{N{\bar{y}}^{2}}}\;+\;{\left[\frac{\text{SE of }b\;}{b}\right]}^{2}$$

and SE of *K* were calculated as $\frac{\text{SE of }b}{b^{2}}$ where *s* ^2^ is the residual mean square of *y* and is the sample mean. Degree day requirements from egg to adult (one generation) and each life stage were also calculated using an average minimum temperature threshold across life stages to establish practicable degree day requirements for phenological modeling.

#### 2.3.2 Adult flight phenology and biofix prediction

Seasonal adult flight phenology of *A. ipsilon* migrant adults was analyzed with nonlinear logistic regression models using generalized least squares in the *nlme* package (25). Adult male counts in sex pheromone baited traps were used as a proxy for adult *A. ipsilon* populations. All site years included in the study had >20 total seasonal *A. ipsilon* trap captures across respective sampling windows. Phenological models were developed using daily minimum and maximum temperatures and three common degree day calculation methods, including simple average, single triangle, and single sine (26–28). Daily land surface temperature data was extracted from PRISM gridded rasters in 800 × 800 m resolution using the *prism* package (37). Adult count data was converted to the seasonal cumulative proportion of trap catch at each site-year location. Similar to Dorman et al. (10), a three-parameter nonlinear regression model was used to predict the cumulative proportion of *A. ipsilon* trap catch (*Y_ij_*) at accumulated growing degree days (hereafter, ‘GDD’) GDD*_ij_* and GDD method *_i_* is


$$Y_{ij}\;=\;a_{i}\;/\;\left(1\;+\;e^{\left[-\left(b_{i}\;-\;x_{ij}\right)\;/\;c_{i}\right]}\right)\;+\;\epsilon_{ij}$$

Where *a* is the upper asymptote of *Y_ij_*, *b* is the value of *x* at which *Y_ij_* equals half its asymptotic value, and *c* is a growth rate parameter. Phenological variation across year*_ij_* was accounted for using variance structure


$$\epsilon_{ij\;}\;=\;N\;\left(0,\sigma^{2}\times {\left|\left|{\text{year}}_{ij}\right|\right|}^{2\delta_{j}}\right)$$

to allow within-group errors to be heteroskedastic. A range of degree day accumulation start dates were evaluated from 1 January to 5 May in five-day increments [i.e., day of year (hereafter, ‘DOY’) 1 to 125], totaling 75 candidate models across the three degree day calculation methods evaluated. The accuracy and performance of candidate models were compared using mean absolute error (MAE), root mean squared error (RMSE), and pseudo coefficient of determination (*R* ^2^) metric


$$R^{2}\;=\;1\;-\;\left[\sum \;{\left(y\;-\;\hat{y}\right)}^{2}\;/\;\sum \;{\left(y\;-\;\bar{y}\right)}^{2}\right]$$

that estimates the fit of nonlinear regression models (29, 30). The average minimum temperature threshold (*t*) for the combined dataset (across diet types) in the temperature-dependent development experiment (9.8°C) was used for all model calculations. Optimal models for each degree day calculation method (simple average, single triangle, single sine) were cross-validated by randomly splitting the data by year into ‘test’ and ‘train’ datasets. Model predictions based on the ‘train’ data were evaluated against ‘test’ observations using the *car* package (31). Simple linear regression was used to evaluate the accuracy and model fit of predictions compared to observed values across degree day calculation methods (with equal minimum temperature threshold and degree day start date parameters) using RMSE and *R* ^2^ metrics, respectively, in the *plm* package (32).

## 3 Results

### 3.1 Temperature-dependent development

**Figure 2:**
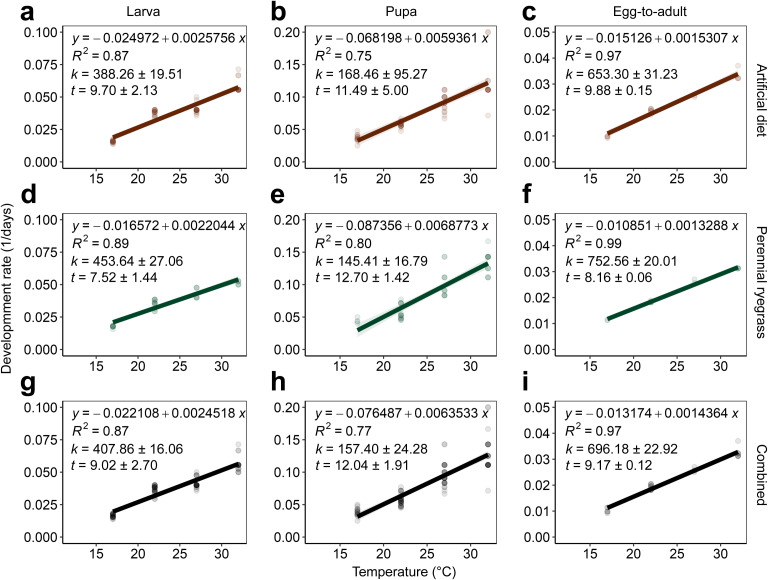
Linear regression analysis of development rate (1/days) and temperature (17–32°C), estimated lower developmental thresholds (*t* ± SE), and thermal requirements in degree-days (*k* ± SE) of *A. ipsilon* life stages (larva, pupa, and egg-to-adult) reared on artificial **(A-C)** and perennial ryegrass diets **(D-F)** and combined datasets across diet treatments **(G-I)**.

For temperature-dependent development experiments, complete larval mortality was observed in the 12°C temperature treatment for both diet types (larvae did not develop beyond second instar) and excluded from regression analyses; mortality in other temperature and diet treatment combinations ranged from 10 to 35%. The number of days required for the development of each life stage was inversely related to temperature, and development rates across life stages were linear from 17 to 32°C for both diet types (**Figure 2**). Across diet types, the number of days required for development was significantly different across temperature treatments for all life stages including egg (*F* = 102.78, *P*< 0.001), larval (*F* = 728.88, *P*< 0.001) and pupal development (*F* = 114.51, *P*< 0.001). For egg development, the number of days for development was not different between 27 and 32°C temperature treatments across diet types (*P<* 0.09); all other treatments were different. Differences were observed across all temperature treatments for larval and pupal life stages for combined data (artificial and perennial ryegrass diet). *Agrotis ipsilon* larvae developed faster on perennial ryegrass compared to artificial diet, averaging 30.62 ± 1.75 and 33.06 ± 2.27 development days for the perennial ryegrass and artificial diet treatments, respectively (*F* = 8.63, *P* = 0.004); differences in development rate were not observed across diet types for pupal development (*F* = 3.47, *P* = 0.07), averaging 12.67 ± 0.91 and 15.66 ± 0.97 for the perennial ryegrass and artificial diet, respectively.

Variable thermal constants (*k*) and lower developmental thresholds (*t*) were observed across diet treatments and *A. ipsilon* life stages (**Figure 2**). Due to insufficient egg laying across temperatures (17 to 32°C) for adult moths reared on perennial ryegrass and artificial diet, *k* and *t* requirements were calculated for the egg life stage with combined data across diet treatments (*y* = –0.01192 + 0.00394*x*, *k* = 96.81 ± 6.08, *t* = 8.05 ± 0.20). *Agrotis ipsilon* thermal requirements in degree-days were standardized across life stages and diet treatments using a mean lower developmental threshold (hereafter, ‘LDT’) (*t* or LDT = 9.8 ± 0.80°C) to estimate degree day accumulation thresholds. Thermal requirements in degree-days (LDT = 9.8°C) for the larval and pupal life stages were lower for perennial ryegrass diet-reared *A. ipsilon* (larva: *k* = 315.92 ± 18.85, pupa: *k* = 178.64 ± 20.62) compared to artificial diet (larva: *k* = 384.08 ± 19.30, pupa: *k* = 193.22 ± 109.27) (**Table 1**). The total thermal requirement to complete one generation (egg-to-adult) were *k* = 601.98 ± 16.01 and *k* = 658.71 ± 31.49 for perennial ryegrass and artificial diet treatments, respectively (**Table 1**). Thermal requirements for combined datasets (across perennial ryegrass and artificial diet treatments) for the egg, larva, pupa, and egg-to-adult life stages were 75.72, 372.44, 186.67, 648.47 degree days, respectively (**Table 1**); small (first to third instar) and large larvae (fourth to sixth instar) required approximately 142.16 and 230.28 degree days, respectively.

**Table 1:** Thermal requirements (*k* ± SE) in degree-days of *A. ipsilon* life stages reared on artificial and perennial ryegrass diets and combined datasets using a constant lower developmental threshold averaged across life stages (LDT or *t* = 9.8°C).

Life stage	Diet type		
	Artificial	Perennial ryegrass	Combined
Egg	–	–	75.72 ± 4.75
Larva	384.08 ± 19.30	315.92 ± 18.85	372.44 ± 14.67
Pupa	193.22 ± 109.27	178.64 ± 20.62	186.67 ± 28.80
Egg-to-adult	658.71 ± 31.49	601.98 ± 16.01	648.47 ± 21.35

### 3.2 Adult flight phenology and biofix prediction

Across degree day calculation methods, the optimal start date for degree day accumulation to predict *A. ipsilon* flight phenology was DOY 120 (i.e., 29–30 April), which had the lowest MAE value for single triangle and marginal improvement >120 for simple average and single sine methods (**Figure 1B**); these results were corroborated with other model fit metrics (RMSE and R^2^) (**Figures 1C, D**). Selected parameters for phenology model validation included a degree day accumulation start date of DOY 120 and LDT = 9.8°C; given the temperate climate of the study region and low mortality of *A. ipsilon* at 32°C in temperature-dependent development experiments, an upper developmental threshold (UDT) was not included in accumulated GDD calculations. Model validation using simple linear regression to evaluate performance of predicted against observed values was significant across degree day calculation methods; single sine (RMSE = 0.177, R^2^ = 0.73, *y* = –0.175 + 0.711*x*) performed marginally better than single triangle (RMSE = 0.178, R^2^ = 0.72, *y* = –0.177 + 0.707*x*) and simple average methods (RMSE = 0.181, R^2^ = 0.71,*y* = –0.180 + 0.700*x*) (**Figures 1E–G**). Nonlinear regression phenology models predicted median *A. ipsilon* flight (*y*_0.50_) at 257.2 (± 95% CI = 240.3–278.1), 269.7 (± 95% CI = 255.1–286.7), and 277.3 (± 95% CI = 263.1–293.7), degree days for simple average, single triangle, and single sine calculation methods, respectively (**Figures 1H–J**).

## 4 Discussion

Across life stages, *A. ipsilon* exhibited a linear development rate from 17 to 32°C temperatures for both diet types evaluated. Survival and development rates were optimal between 27 and 32°C; previous research assumed development rates >30°C were nonlinear, albeit temperatures greater than 30°C were not evaluated (16, 20). Additional research is needed to determine the precise upper developmental threshold for this species across life stages currently speculated to be ~36°C (38).

Similar to previous research investigating *A. ipsilon* development on maize and artificial diet, differences in development rate were detected across diet types in the present study (16). However, research by Santos and Shields (16) observed slower development when *A. ipsilon* was reared on a maize diet compared to an artificial diet, which was likely attributed to maize being a ‘suboptimal’ food source, prolonging development and requiring additional larval instars before pupation. In contrast, *A. ipsilon* development rate was faster on the perennial ryegrass diet compared to artificial diet; however, differences in the number of larval instars required to reach pupation were not detected across diet treatments (six instars were observed for both diet types). Considering a faster development rate is likely advantageous evolutionarily to evade predation and maximize reproduction potential, the results of the temperature-dependent experiments in the present study suggest grass seed crops are likely a preferred host and suitable for meeting the nutritional requirements needed to complete development as well as the metabolic reserves required for autumn migrations southward to overwintering habitat (16, 21).

The mean lower developmental threshold (LDT) across combined datasets (i.e., *A. ipsilon* reared on perennial ryegrass or artificial diet) for complete development from oviposition to adult eclosion was approximately 9.8°C, which is slightly lower than previous studies reporting LDT estimates of 10.4°C and 10.7°C for this species (20, 33–35). To determine practical parameters for spatiotemporal phenology forecasting, an LDT of 9.8°C was used to calculate thermal requirements across life stages and diet types (**Table 1**). Similar to Luckmann et al. (20), that found 52, 353, 238, and 643 degree days (LDT = 10.4°C) were required for egg, larvae, pupae, and oviposition to adult eclosion, respectively, we estimated similar thermal requirements across diet types, including 76, 372, 187, 648 degree days (LDT = 9.8°C) for the same life stage intervals. Other studies determined degree day requirements for *A. ipsilon* to complete one generation were 643 and 692 (LDT = ~10.7°C) (33–35). In the present study, the thermal requirements for *A. ipsilon* to complete one generation reared on perennial ryegrass was approximately 602 compared to 659 for *A. ipsilon* reared on an artificial diet. These results demonstrate the utility of refining phenology models with pest development parameters on primary crop hosts within the spatial extent of phenological forecasts to improve the accuracy of model predictions. Furthermore, an LDT of 9.8°C in the present study suggests physiological stress and limited overwintering potential (as diapausing pupae) or continual reproductive development in geographic zones with sustained temperatures below this temperature threshold. It is speculated that during the winter months in the Northern Hemisphere, the current northern extent of *A. ipsilon* winter-breeding populations emigrating southward is below 33°N latitude; warmer soil and land surface temperatures due to climate change may fuel range expansion of overwintering populations to more northern latitudes altering the timing and intensity of spring immigration events in Oregon (15, 38).

The phenological models developed to predict the seasonal median flight of *A. ipsilon* migrants in Oregon performed similarly well when validated against new data (i.e., after partitioning the data into ‘train’ and ‘test’ datasets randomly across sample years). The optimal date to start degree day accumulations was 29–30 April (DOY 120), and marginal differences were detected in model performance across degree day calculation methods (simple average, single triangle, single sine), albeit single-sine was the most accurate (lowest RMSE value). The timing of median flight migrations of *A. ipsilon* from southern winter-breeding locations was reliably predicted using land surface temperature alone when models were trained and validated with field data spanning over two decades, providing an adequate assessment of interannual variability in *A. ipsilon* migration events across the study region. More precise predictions may be achieved by incorporating additional environmental predictors often associated with migratory noctuid flights and *A. ipsilon* migration, in particular, including photoperiod, minimum and maximum temperatures in the source region and along migratory routes, as well as the trajectory of low-level jet stream winds using the NOAA (National Oceanic and Atmospheric Administration) Air Resources Laboratory’s Hybrid Single-Particle Lagrangian Integrated Trajectory model (HYSPLIT), speculated to be a significant predictor of the timing and intensity of noctuid migration events (36, 38, 39). The simplicity of predictive models that achieve reasonably accurate results to produce coarse areawide guidelines on biological monitoring sampling windows (optimal timing to assess pest population densities in crop production systems) to improve within-season decision-making may be preferable for researchers and field practitioners to utilize models for developing efficient and real-time decision support applications for field use. The complex population dynamics and migratory patterns of *A. ipsilon* and other noctuid pests are likely to change in unpredictable ways with unusual weather events and climate change; as such, continual surveillance and model calibration should be exercised to ensure model prediction accuracy across the Willamette Valley and central Oregon in future years. Implementation of phenological forecasts and similar spatiotemporal areawide projections of endemic and invasive agricultural pest populations are critical for improving pest surveillance; however, models alone are not sufficient for providing management decisions to growers independent of field-scale biological monitoring assessments to ensure appropriate management actions are warranted.

## 5 Conclusion

Findings from temperature-dependent development experiments and modeling of *A. ipsilon* seasonal flight in the present study can be incorporated into a general phenology model (i.e., vegetable and other host crops using combined data results) and grass seed crop-specific model using the predicted median flight as the biofix of *A. ipsilon* adult arrival and oviposition (egg-laying) to initiate degree day accumulation for subsequent life stages. Furthermore, the model parameters presented can be incorporated into a decision support platform that shares real-time areawide interpolation risk maps of *A. ipsilon* life stages across time and space using high resolution (e.g., 800 × 800 m PRISM data) gridded weather data (**Figure 1K**). Space-time forecasts of priority pests using an areawide pest management framework are critical for coordinating pest surveillance and management resources to promote sustainable pest management practices and achieve optimal pest suppression (population densities below economic concern) while reducing the environmental footprint and economic input costs associated with pesticide overuse.

## Data Availability

The raw data supporting the conclusions of this article will be made available by the authors, without undue reservation.
